# Biomembranes Based on Potato Starch Modified by Dry Heating Treatment: One Sustainable Strategy to Amplify the Use of Starch as a Biomaterial

**DOI:** 10.1021/acs.biomac.4c01294

**Published:** 2025-02-24

**Authors:** Pedro Augusto Invernizzi Sponchiado, Maryanne Trafani Melo, Juçara G. Cominal, Milena Martelli Tosi, Pietro Ciancaglini, Ana Paula Ramos, Bianca Chieregato Maniglia

**Affiliations:** † Department of Physical Chemistry, Institute of Chemistry of São Carlos (IQSC), University of São Paulo (USP), São Carlos, São Paulo 13566-590, Brazil; ‡ Department of Chemistry, Faculty of Philosophy, Sciences and Letters at Ribeirão Preto (FFCLRP), University of São Paulo (USP), Ribeirão Preto, São Paulo 14040-901, Brazil; § Department of Food Engineering, Faculty of Animal Science and Food Engineering, Postgraduate Programme in Materials Science and Engineering, University of São Paulo, 13635-900 Pirassununga, São Paulo, Brazil

## Abstract

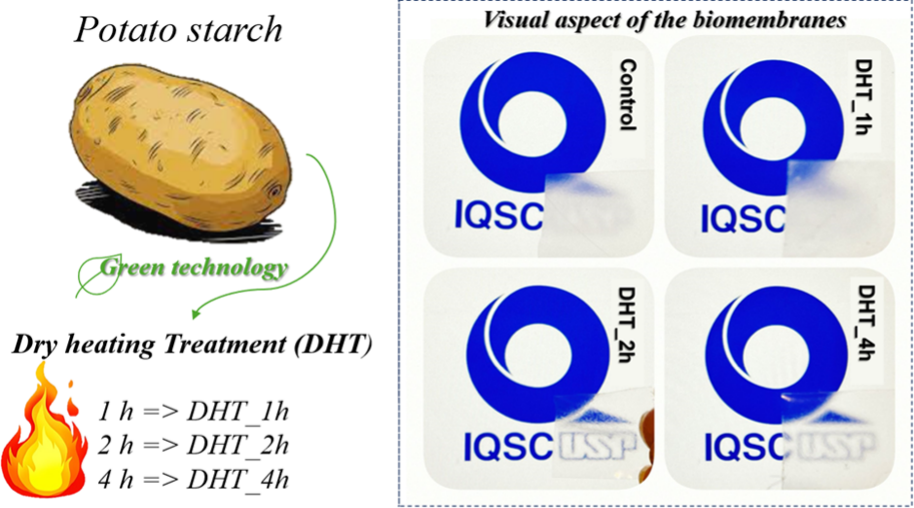

The exceptional biocompatibility of polymeric membranes drives their use in biomaterials, but structural modifications are needed to improve their mechanical properties. This study investigated dry heating treatment (DHT) as an ecofriendly and cost-effective approach to modifying potato starch for biomembrane fabrication. DHT-treated starch (2 h) produced biomembranes with a denser structure, smoother surfaces, and significantly improved mechanical properties, including higher tensile strength (∼6×), rigidity (∼15×), and relative crystallinity (∼2×) while reducing flexibility (∼5×), compared to native starch membranes. These membranes also exhibited lower moisture content, reduced hydrophilicity, higher surface energy, decreased biodegradability, and enhanced bioactivity, as shown by hydroxyapatite formation in simulated body fluid. Importantly, they were nontoxic to osteoblasts, emphasizing their potential for medical applications. This study highlights DHT as a sustainable and innovative method for modifying starch to develop advanced biomaterials for medical applications.

## 1 Introduction

Organic biomembranes are typically derived from natural polymers like chitosan, cellulose, alginates, and collagen or from synthetic polymers such as polyurethane, poly(ethylene glycol) (PEG), poly(acrylamide), polylactic acid, polysulfone, and poly(*N*-vinyl-2-pyrrolidone).^1−6^ Starch is a natural, nontoxic, and cost-effective biodegradable polymer with excellent biocompatibility. It is derived from abundant and renewable natural sources, making it an environmentally sustainable material.

Nonetheless, it is widely acknowledged that the performance of native starch is suboptimal, and the properties of starch-based plastics fall short when compared to those of conventional counterparts. To enhance the performance of native starch, various modifications have been explored, and among them, dry heating treatment (DHT) emerges as a compelling alternative with potential applications across different industries. This treatment yields materials with improved mechanical and functional properties such as barrier and wettability.^7^ DHT of starch stands out as the optimal choice in the current landscape, primarily due to its ability to sidestep environmental hazards and potential risks to human health. Moreover, it proves to be a cost-effective alternative when compared to other modification methods.^8^ This approach not only circumvents the generation of environmental waste but also ensures the absence of chemical residues, making the modified starch suitable for use in both food and medical applications.^8^

This dual advantage positions DHT as a superior and sustainable method for starch modification.^9^ According to literature findings,^10−13^ DHT induces partial substitution of hydroxyl groups with carbonyl groups, fostering new intermolecular associations. Additionally, DHT results in depolymerization, causing reduction in the molecular size, especially affecting molecules of intermediate size, forming distinct groups with larger and smaller molecules. This adjustment in molecule sizes enhances reassociation tendencies, favoring short- and long-term retrogradation.^7^ Importantly, the literature also notes that the polymer structure, including polymer chain length and associated functional groups, plays a significant role in influencing the biodegradation process.^14^ Additionally, each starch source reacts uniquely to DHT. In this sense, potato starch possesses distinct properties that require a specific evaluation during DHT processing. Potato starch is distinguished by its long amylopectin and amylose chain lengths, large particle size, and the phosphate ester groups presence, which influence the final properties of the biomembranes compared to other sources.^15,16^

In thebiomedical area, membranes can be used in the development of artificial organs (oxygenator, pancreas, and artificial liver) to increase the ideal functionalization of the physiological functions of the organs,^17,18^ increase the capacity for possible tissue repair and regeneration/injured organs,^19−23^ as a membrane-based drug delivery system,^24^ or as different molecules of separation interest such as antibiotics^25−27^ or proteins.^28^ Developing polymeric biomembranes capable of orchestrating the intricate cascade of events in tissue regeneration necessitates faithful reproduction of the native tissue composition and structure. Beyond providing a biochemical stimulus, these polymeric membranes must furnish mechanical support, isolate, safeguard the defect site, and stimulate tissue repair at a controlled biodegradation rate. Importantly, this should be achieved without necessitating additional removal surgery after the treatment.^29,30^ It makes starch a promising candidate for the creation of functional biomedical materials.^31^ Recent research has highlighted starch’s positive impact on cell attachment, proliferation, and differentiation, affirming its supportive role in cellular activity during the formation of a new extracellular matrix.^31−33^ In particular, biopolymers such as proteins (fibrin gels, silk, collagen, and soy) and polysaccharides (alginate, cellulose, hyaluronic acid derivatives starch, and chitin/chitosan) have been applied in the development of biomembranes for guided bone regeneration (GBR), aiming to improve bone tissue regeneration and healing outcomes.^34^

Therefore, this study investigated the potential of potato starch modified by DHT using different periods of time to produce biomembranes, focusing on obtaining biomaterials with potential use as GBR that should present adequate biodegradability and good mechanical and bioactivity. In the literature, the use of DHT has been applied to produce modified starch focusing mainly on the food sector.^35−37^ In this sense, this work brings one innovative purpose to explore DHT as one alternative to modify starch to produce a biomaterial, specifically biomembranes. Finally, it is also worth highlighting the benefits of this green approach (DHT), which can be translated in an innovative way for medical applications.

## 2 Materials and Methods

### 2.1 Material

Cargill Agricola, Brazil, supplied potato starch with a moisture content of 11.1 ± 0.20% and an apparent amylose content of 22.6 g/100 g starch (dry basis) and apparent amylopectin content of 77.4 g/100 g starch (dry basis), which was subsequently divided into 50 g portions. These portions were then spread as a powder layer approximately 1.0–1.5 mm thick, placed in 30 × 30 cm aluminum bags, and subjected to DHT in a convective oven (ModFic03, Famo, São Paulo, Brazil) at 130 °C for 1, 2, and 4 h, designated as DHT_1h, DHT_2h, and DHT_4h, respectively, following the modification process outlined in Maniglia et al.^10^ A sample of native potato starch (without modification) was used as a control group. After modification, the starch underwent cooling, grinding, and sieving (60 mesh, 0.250 mm) and was then packaged and stored for future use. All aqueous solutions were prepared using ultrapure, dust-free water (Milli-Q system) with a resistivity of 18.2 MΩ cm. Glycerol (analytical grade) from Sigma-Aldrich (Brazil) served as a plasticizer in the process. All chemical reagents utilized were of analytical grade. The apparent amylose contents of the modified starches DHT_1h, DHT_2h, and DHT_4h were, respectively, 25.3 ± 0.7, 26.9 ± 1.1, and 26.1 ± 1.2 g/100 g starch on a dry basis [measured by the iodine colorimetric method (Juliano, 1971) using standard potato starch (Sigma A0512, Sigma-Aldrich, USA) for calibration]. The apparent amylopectin content was determined by subtracting the amylose content from 100%. In this sense, the amylopectin contents of the modified starches DHT_1h, DHT_2h, and DHT_4h were, respectively, 74.7, 73.1, and 73.9 g/100 g starch on a dry basis.

### 2.2 Biomembranes Elaboration

The membranes were produced using the casting method, following the methodology of Silva et al.^36^ A suspension of potato starch (5 wt % on a dry basis) was heated to 85 °C with magnetic stirring for 45 min in a jacketed beaker connected to a circulating water bath. Afterward, glycerol (25% of the potato starch weight) was added, and the mixture continued to heat at 85 °C for an additional 15 min with stirring. To remove air bubbles, the suspension was placed in an ultrasound bath for 10 min at room temperature. The resulting mixture was transferred to acrylic Petri dishes (0.15 g/cm^2^) and dried in a climatic chamber [35 °C and 45% relative humidity (RH) for 10 h]. Finally, the biomembranes were removed from the dishes and conditioned in desiccators with a saturated NaBr solution (58% RH) for at least 48 h before characterization. The biomembranes were classified as control (based on native potato starch), DHT_1h, DHT_2h, and DHT_4h (based on potato starch modified by DHT for 1, 2, and 4 h).

### 2.3 Characterization of the Biomembranes

#### 2.3.1 Thickness

The thickness of the biomembranes was assessed by measuring six distinct areas using a digital micrometer (MITUTOYO, Japan) with a precision of 0.001 mm. The average value is regarded as the final thickness.

#### 2.3.2 Mechanical Properties

The tensile test adhered to ASTM Method D882-12^39^ involving an average of 10 measurements for each case. Elongation at break (*E*) and tensile strength (TS) were measured using a texture analyzer (TAXT2i Stable Micro Systems, UK) equipped with a 50 kgf (490 N) load cell. The biomembranes were cut into strips (2.54 cm of width and a minimum length of 10 cm). The initial distance between the grips was set at 80 mm, and the crosshead speed was fixed at 1.0 mm/s. The Young’s modulus (YM) was determined from the slope of the initial linear segment of the stress–strain curve, using Texture Expert V.1.22 software (SMS).

#### 2.3.3 Morphology

The morphology of the biomembranes was examined by using atomic force microscopy (AFM) and scanning electron microscopy (SEM).

The biomembranes were analyzed with AFM (Multimode 8, Bruker, UK) at a scan rate of 1 Hz. Before testing, the biomembranes were cut into pieces and mounted on double-sided tape. The AFM scanning area was 10 μm × 10 μm, and image analysis was performed with Nanoscope Analysis 1.50 software (Bruker, UK). Eqs 1 and 2 were used, respectively, to calculate the arithmetic mean of the surface height deviations (*R*_a_) and the root-mean-square average of height deviations (*R*_q_).
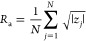
1
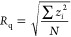
2

The peak-to-valley height difference within the analyzed region is represented by *z*, and *N* refers to the number of points within the box cursor during image acquisition.

Additionally, the surface of the gold-coated biomembranes was analyzed using a scanning electron microscope (Superscan SS-550, Shimadzu, Japan) with magnifications of 100×, 240×, and 1000× (voltage of 15 kV).

#### 2.3.4 Structural Characterization by X-ray Diffraction

X-ray diffraction (XRD) analysis of the membranes was performed using an X-ray diffractometer (Siemens D5005 model, Baden–Württemberg, Germany) with 2θ values ranging from 2 to 60°. The scan rate was set to 0.02°/min, with a voltage of 40 kV and a current of 30 mA. The relative crystallinity (RC) was determined following the method outlined by Nara and Komiya,^38^ considering 2θ values from 2 to 60°. The analysis was performed using Origin software, version 9.6.5 (Microcal Inc., Northampton, MA, USA).

#### 2.3.5 Thermal Properties by a Differential Scanning Calorimeter

The thermal properties of the biomembranes were assessed using a differential scanning calorimeter (DSC TA2010, TA Instruments, New Castle, DE, USA). The biomembranes were placed in hermetically sealed aluminum TA pans and subjected to two heating cycles from −60 to 100 °C at a rate of 5 °C/min under a nitrogen atmosphere (45 mL/min). An empty aluminum pan was used as the reference. The DSC cell was cooled with liquid nitrogen before each heating cycle. The melting temperature (*T*_m_) and melting enthalpy (Δ*H*_m_) were determined from the thermal curves by using Universal Analysis V1.7F software (TA Instruments, New Castle, DE, USA).

#### 2.3.6 Opacity

The opacity of the biomembranes was assessed in triplicate with a colorimeter (HUNTERLAB, model ColorQuest XE, USA) and determined using the relation between the opacity of the biomembrane superposed on the black standard (*Y*_b_) and the opacity of the biomembrane superposed on the white standard (*Y*_w_), following eq 3.
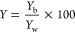
3

#### 2.3.7 Moisture Content

The moisture content of the membranes was assessed using the oven-dry method. Briefly, 2.0 g of films was subjected to drying at 105 °C until a constant weight was achieved. The outcomes were presented as a percentage of dry weight with the average ± standard deviation (SD), in triplicate (*n* = 3).

#### 2.3.8 Wettability and Surface Free Energy

The wettability and surface free energy of the membranes were assessed using an optical contact angle (θ) (DataPhysics OCA20, Germany). The biomembranes were affixed to the surface of a glass slide using tweezers and double-sided tape and then positioned on a horizontally movable stage. A motor-driven syringe introduced three different standard liquids (deionized water, diiodomethane, and formamide) at 25 °C.

Images were captured by a camera, and the contact angle of the film was recorded after 10 s. The surface free energy and its components [dispersive (γ^d^) and polar (γ^p^)] were calculated using the Owens–Wendt method^39^ as eq 4. Referring to *S* and *L*, they denote the solid and liquid surfaces, respectively.

The calculation of the surface free energy proceeded as follows
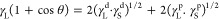
4

#### 2.3.9 Biodegradability

The membranes, shaped into circles measuring 6.25 cm^2^, were positioned in 6-well cell culture plates and submerged in 5 mL of the cell culture medium (α-MEM, Gibco) for 8, 24, 48, and 72 h at 37 ± 2 °C in an environment containing 5% CO_2_. Subsequently, the membranes were retrieved and subjected to drying in an oven until they reached complete dryness. Biodegradability was determined as the percentage of dry matter remaining on the membrane after immersion in the cell culture medium at the intervals 24 and 72 h.

A curve-fitting model was employed to determine the rate constant for biomembrane biodegradation over time. Conducted in batch mode, this process utilized a dynamic mass balance for biomembrane degradation following the first-order reaction model as represented in eq 5.
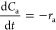
5where *C*_a_ (g/L) is the mass of the biodegraded biomembrane at time *t* (hours), and −*r*_a_ (g/L·h) is the rate of biomembrane biodegradation in the cell culture medium.

The biodegradation profile was fitted using a first-order reaction model, and the time required to achieve complete biodegradation (100%) was estimated.

#### 2.3.10 Bioactivity in Simulated Body Fluid and Cytotoxicity of the Membranes to Osteoblasts

Bioactivity assays are widely recognized methods for anticipating the biological performance of a substance. The bioactivity can be studied by the capacity of a material to induce hydroxyapatite (HAp) precipitation postimmersion in simulated body fluid (SBF).^40^ The formulation and preparation details of the SBF were previously elucidated by Kokubo and Takadama.^40^ In the in vitro experiments, 2.5 × 2.5 cm pieces were excised and immersed in 10 mL of SBF at 37 °C for 30 min. After SBF exposure, the membranes underwent a thorough rinse with ultrapure water to eliminate soluble material not specifically adsorbed on the membrane, followed by air-drying at room temperature in a sealed container. Ultimately, the formation of the apatite layer was scrutinized by assessing alterations in the surface morphology of the films through SEM and XRD analyses.

For the cell cultures, preosteoblast cells from the murine lineage MC3T3-E1 (American Type Culture Collection-ATCC CRL-2593) were cultured in a minimum essential medium (α-MEM, Gibco), supplemented with 10% fetal bovine serum and 1% (v/v) streptomycin/penicillin. Upon reaching confluence, the cells were trypsinized, resuspended in α-MEM, and then seeded at a density of 5 × 10^4^ cells/well on starch membrane discs placed in 24-well plates. The plates were subsequently incubated at 37 °C in an atmosphere containing 5% CO_2_. To induce osteogenesis, ascorbic acid and β-glycerophosphate, known for promoting cell differentiation, were added to the medium. The culture medium was refreshed approximately two to three times per week.

*Cell viability* was assessed using the MTT assay following the methodology outlined by Faria et al.^41^ In brief, the membranes, incubated in the cell culture for 24 and 72 h under conditions of 37 °C and 5% CO_2_, were treated with 1.0 mg/mL of MTT (3-(4,5-dimethylthiazol-2-yl)-2,5-diphenyltetrazolium) and incubated under the same conditions for an additional 4 h. This process produced a highly colored compound (formazan) reflecting cellular dehydrogenase activity upon reduction with NADH. After incubation, formazan crystals were dissolved in 2-propanol, and absorbance was measured at 560 and 690 nm using a spectrophotometer (Multi-Mode Microplate Reader, SpectraMaxM3, USA) to determine the mitochondrial dehydrogenase concentration. Cell viability was expressed as a percentage relative to the average of three experiments, compared to the control (native starch-based biomembranes) for each analyzed period.

### 2.4 Statistical Analysis

The data were analyzed statistically using analysis of variance (ANOVA), followed by Tukey’s test for mean comparison at a 95% confidence interval (*p* < 0.05). Statistical analysis was conducted using the STATISTICA 7 software.

## 3 Results and Discussion

### 3.1 Crystallinity by XRD and Thermal Properties by DSC

**Figure 1:**
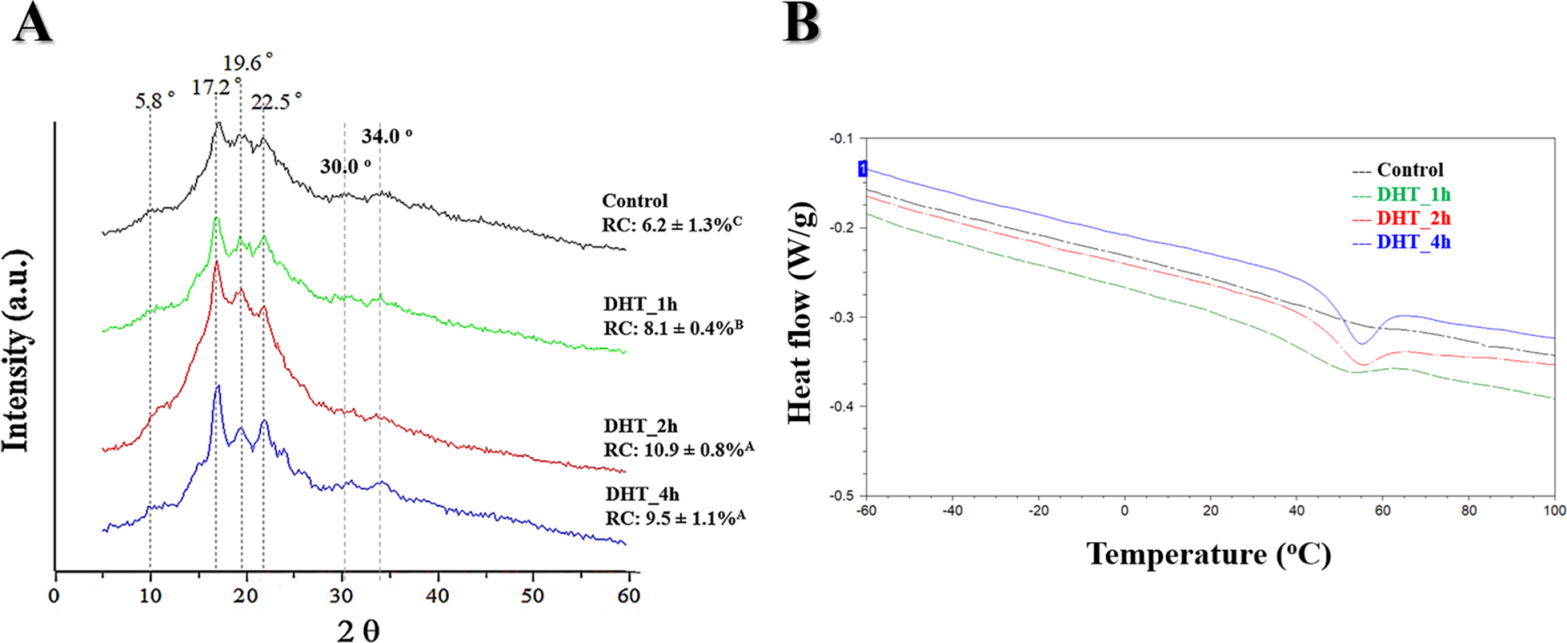
(A) XRD patterns of the biomembranes based on nonmodified (control) and modified potato starch by DHT (1, 2, and 4 h) and their respective RC. A–C: Distinct letters denote a statistically significant difference among the four biomembrane compositions (Tukey test, *p* < 0.05). (B) DSC thermograms of the biomembranes based on nonmodified (control) and modified potato starch by DHT (1, 2, and 4 h).

In Figure 1, X-ray diffractograms depict the comparison between nonmodified and dry heating-treated biomembranes. A semicrystalline characteristic, characterized by the presence of amorphous and crystalline regions, is evident. Peaks at 5.8, 17.2, 19.6, 22.5, 30.0, and 34.0° (2θ) are observed. We can observe that the intensity of the peak at 17° is more pronounced for the modified starch biomembranes, highlighting to DHT_2h. The formation of the film matrix comes from the recrystallization of amylose, mainly when there is high water mobility within the starch matrix.^42^ Amylopectin may also play a role in influencing this process during film formation, albeit at a slower rate and under conditions of high RH, typically taking several days to occur.^43^ Consequently, the prominent diffraction peak at 17° and smaller peaks at 20 and 22° mainly result from the recrystallization of amylose molecules present in the films. According to Sponchiado,^44^ potato starches modified from DHT underwent a depolymerization process, presenting a higher content of polymers with shorter chains, which contributed to interactions that promoted greater recrystallization, exhibiting more pronounced diffraction peaks at 17 and 22° in comparison with native starch-based biomembranes (control). Furthermore, the low-intensity peaks at 30.0 and 34.5° that were demonstrated for all samples may correspond to the presence of starch nanocrystals promoted mainly by the contribution of amylopectin in the matrix as discussed by La Fuente et al.^45^

In a quantitative assessment, the final degree of crystallinity in the films is influenced by the chain’s ability to form crystals and its mobility. The modified starch biomembranes exhibited higher RC values, which can be attributed to the newly structured matrix formed during the dry-heating process. As observed in the mechanical properties and morphology by SEM and AFM images, the formation of a structure with a smoother surface formed by the modified starches was visible when compared to the native starches, which presented a rougher surface (confirmed by *R*_a_ and *R*_q_ values). When the RC is higher, it indicates a matrix with more ordered crystalline structures and less intermolecular flexibility, and this may be the result of greater interactions between the starch biopolymers, with fewer free hydroxyl sites.^46^ Studies indicate that in films produced from starch modified by acetylation, the incorporation of bulky acetate groups into the polymer structure limits chain mobility, resulting in greater structural restriction.^47^

La Fuente et al.^11^ also observed that films based on DHT-modified cassava starches showed superior RC when compared to the native. For other side, González-Soto et al.^48^ reported that the potato starch modified by dual-modified (acetylation and cross-linking) showed reduction in the RC, and the observed effect was attributed to increased density fluctuations due to a higher number of lattice defects introduced by cross-linking within the crystalline phase. Alternatively, it may stem from changes in local density near the cross-link sites within the amorphous phase.^48^ In this sense, each type of modification can create a different impact in the starches, resulting in different performances in the matrix biomembrane, being therefore one interesting alternative depending on the application.

Therefore, when an application in bone biomaterials is contemplated, the DHT method emerges as a promising alternative to produce modified potato starch. This method has the potential to yield films with commendable mechanical and morphological properties, aligning well with the requirements of such applications.

Table 1 shows the thermal properties obtained from the DSC thermograms (Figure 1B) of the biomembranes based on potato starch (control, DHT_1h, DHT_2h, and DHT_4h). All the samples showed a melting temperature (*T*_m_) and one endothermic peak of fusion (Δ*H*_m_). At the *T*_m_, the starch films undergo structural breakdown because of polymer melting and subsequent reassociation.^49^ The heat (enthalpy) of fusion (Δ*H*_m_), represented by the area beneath the endothermic peak, serves as an indicator of the crystallinity of the polymeric films.^50^ The biomembranes based on modified starch showed super values of *T*_m_ and Δ*H*_m_, indicating that there is a higher network of polymers in the matrix, which corroborates the fact that structures are more organized. It is important to emphasize that all biomembranes exhibited melting temperatures above the physiological temperature of 37 °C. Moreover, the enhanced thermal stability of the starch biomembranes reduces the risks associated with higher temperatures, which can accelerate unwanted chemical reactions or biological degradation, thereby extending the lifespan of the biomaterial.^51^

**Table 1:** Thermal Properties of the Biomembranes Based on Potato Starch (Control, DHT_1h, DHT_2h, and DHT_4h)^a^

samples	*T*_m_ (°C)	Δ*H*_m_ (W/g)
control	49.6 ± 1.2^c^	1.6 ± 0.2^d^
DHT_1h	53.6 ± 0.8^b^	2.5 ± 0.3^c^
DHT_2h	54.8 ± 1.0^b^	3.1 ± 0.3^b^
DHT_4h	65.5 ± 1.3^a^	4.2 ± 0.4^a^

a Average ± SD (*n* = 3). a–d: Distinct letters in the same column denote a statistically significant difference among the four biomembrane compositions (Tukey test, *p* < 0.05). Tm: melting temperature; Δ*H*: melting enthalpy.

### 3.2 Visual Aspect

**Figure 2:**
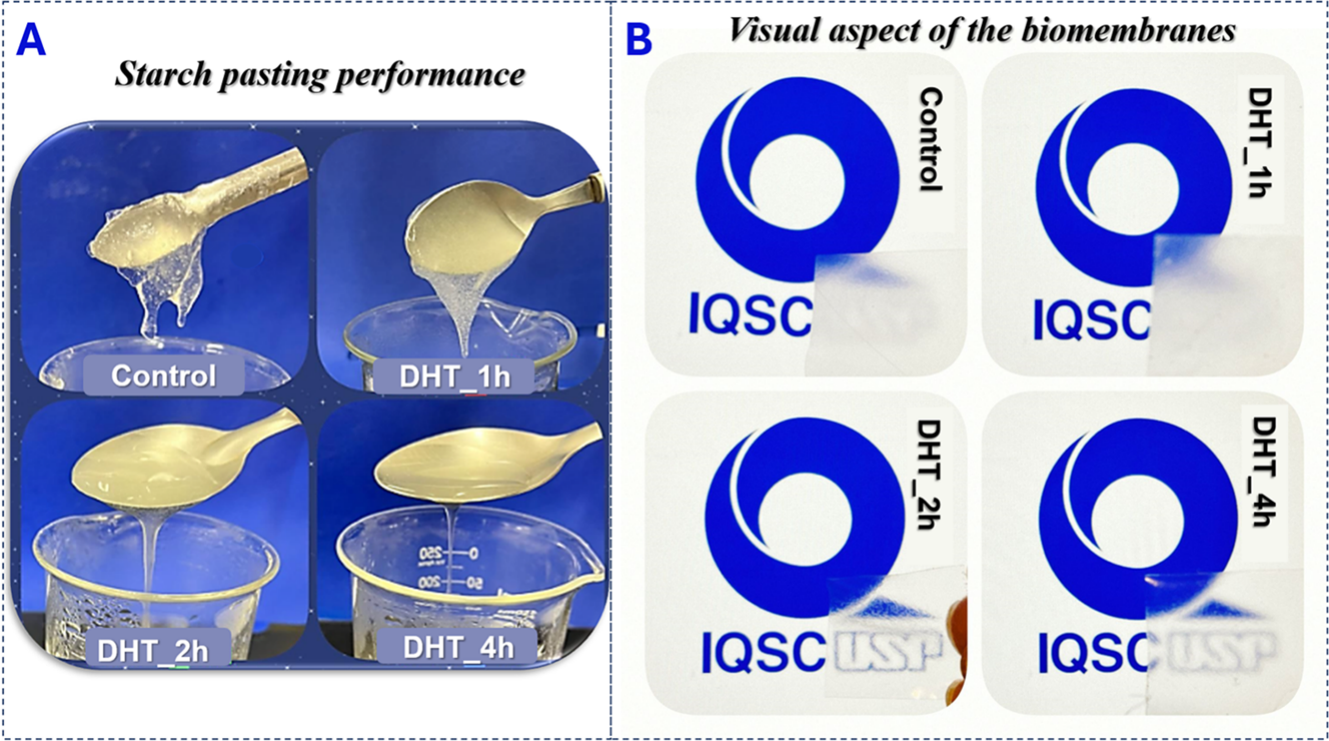
Visual aspect of the (A) starch pasting performance and the (B) membranes based on native (control) and the modified potato starches (DHT_1h, DHT_2h, and DHT_4h).

Figure 2A illustrates the visual pasting performance of native and modified starches (DHT for 1, 2, and 4 h). The DHT changed the pasting performance and visual aspect of the membranes (data of viscosity profile shown by Sponchiado^44^). This may be related to structural modifications of the starch chemical structure, consistent with findings outlined by Sponchiado.^44^ DHT is able to promote oxidation and depolymerization, which are important factors for the pasting and gel performance of these modified starches according to Sponchiado.^44^ The molecular depolymerization weakens the structure of starch granules, resulting in more fluid pastes when DHT is applied longer to potato starch (Figure 2A).

Figure 2B also shows the visual aspect of the biomembranes. Notably, the biomembranes exhibit a nonstick characteristic, making them easily detachable from the acrylic Petri dishes in which they were prepared. In addition, we can observe that biomembranes based on modified starches are less opaque (especially for the DHT_2h biomembrane) than the control based on native potato starch (opacity data are presented in Table 3).

The opacity can be associated with the thickness of the biomembrane, but there was no statistically significant variation observed between the biomembranes’ thickness (*p* > 0.05) (Table 3). The opacity of the materials also can be associated with the organization of the matrix. According to Bertuzzi et al.,^52^ the increase in the crystalline zone decreases the absorbance and increase the film transparence. Therefore, the results indicate that the DHT-promoted modified starches are able to form biomembrane matrices with different spatial organization when compared to the native.

### 3.3 Thickness, Mechanical Properties, and Morphology by SEM of the Biomembranes

The thickness of the biomembranes ranged from 60 to 80 μm (Table 2). Notably, there was no statistically significant differences in the thickness of the biomembranes (*p* > 0.05). The values lower than 0.250 mm categorize these materials as films since membranes thicker than this limit are considered sheets.^11^

**Table 2:** Thickness, TS, Elongation at Break, and YM of the Biomembranes Based on Potato Starch (Control, DHT_1h, DHT_2h, and DHT_4h)^a^

samples	thickness (μm)	tensile strength (MPa)	elongation at break (%)	Young’s modulus (MPa)
control	78 ± 11a	4.68 ± 0.44d	29.41 ± 2.48a	97.48 ± 12.66a
DHT_1h	69 ± 15a	9.96 ± 0.70c	18.07 ± 1.47b	676.60 ± 107.82b
DHT_2h	74 ± 8a	28.52 ± 1.47a	6.26 ± 1.14c	1415.66 ± 151.70c
DHT_4h	82 ± 16a	20.84 ± 2.90b	2.72 ± 0.53d	605.41 ± 142.45b

a Average ± SD (*n* = 10). a–d: Distinct letters in the same column denote a statistically significant difference among the four biomembrane compositions (Tukey test, *p* < 0.05).

**Figure 3:**
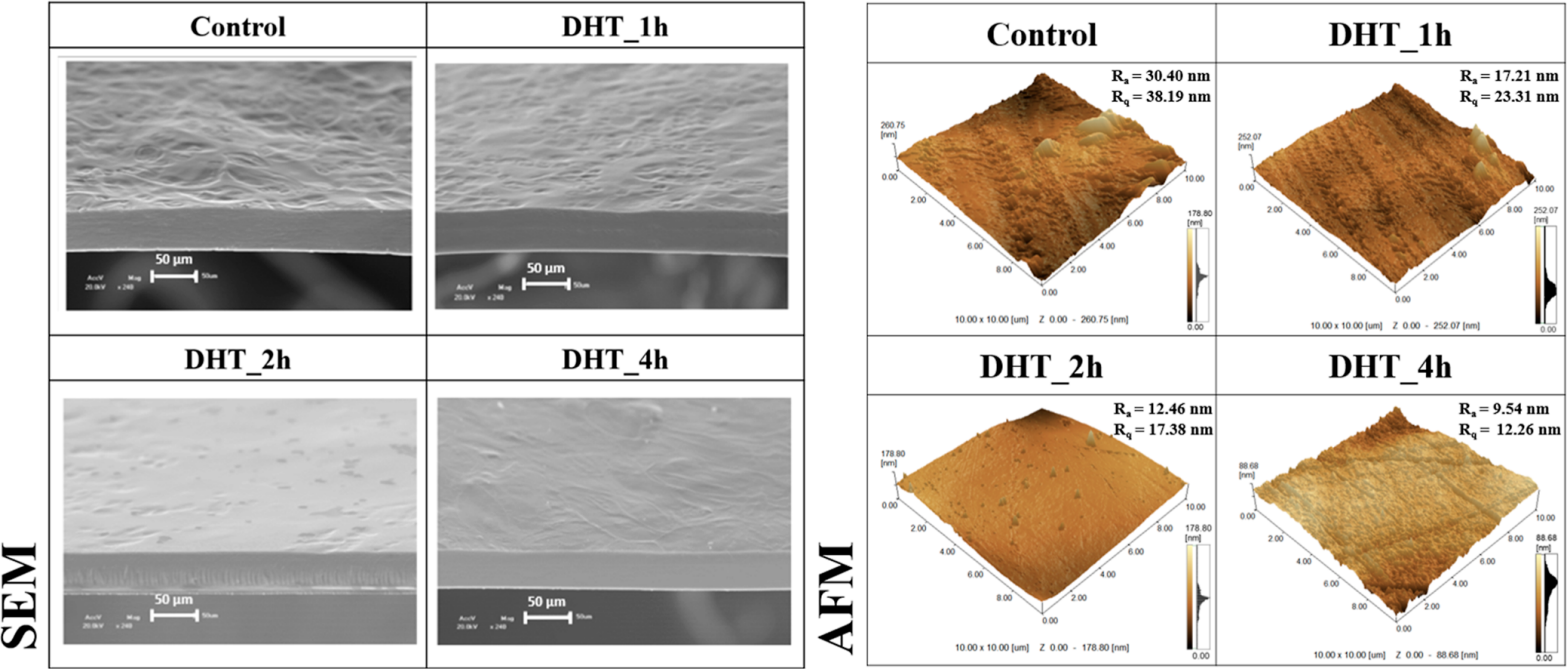
Structural characterization of the biomembranes. Scanning electron micrographs of the cross sections (scale bars correspond to 50 μm images) and AFM images and roughness parameters of the biomembranes based on potato starch (control, DHT_1h, DHT_2h, and DHT_4h).

Figure 3 depicts the morphology of the biomembranes assessed by SEM and AFM images with roughness values (average and root-mean-square). The SEM images reveal distinct characteristics when comparing modified and nonmodified starch (control) biomembranes. The modified biomembranes exhibit a dense structure and sleek surfaces, contrasting with the nonmodified starch biomembranes, and it was more visible for DHT_2h and DHT_4h. The same improvement in the homogeneity of the surface was observed for films based on cassava starch modified by ozone or DHT.^11,53^ Sponchiado^44^ highlighted that DHT causes oxidation and depolymerization in the potato starch biopolymers, resulting in a reduction in the crystalline portion of the granule. It indicates that DHT promotes changes in molecular charges and chemical affinity between the starch biopolymers. These alterations influence intermolecular interactions, thereby modifying the film morphology. In addition, a notably smooth surface in the cross-section is apparent and visible for all biomembranes, with no visible cracks, likely formed during film breakage. In the SEM images, it was not possible to visualize any nongelatinized starch granule in the polymeric matrix as observed by La Fuente et al.^11^ for films based on cassava starch modified by DHT (130 °C—4 h), indicating that the biomembrane elaboration process carried out in this study was effective since it did not present visible nongelatinized starch grains (smooth surface in cross section), cooperating for the formation of the biomembrane matrix.

Considering the AFM images, the roughness values were higher for the control biomembranes compared to those for the modified starch biomembranes. Specifically, the average roughness (*R*_a_) and the root-mean-square roughness (*R*_q_) were lower for the DHT_2h and DHT_4h biomembranes. These findings are aligned with the results observed by the SEM images. It indicates that the SEM and AFM results indicated that DHT could effectively affect the morphology of starch films, contributing to the outstanding properties of biomembranes.

In relation to the mechanical properties, there was one improvement in the TS and reduction in the elongation at break with the increase of the DHT period. Moreover, the impact of DHT on YM was extremely significant, revealing that biomembranes derived from modified starches were around 6 (DHT_1h or DHT_4h) to 14 times (DHT_2h) higher than their counterparts based on native starch. The results indicate that the DHT of potato starch produced stronger biomembranes, and 2 h of DHT was the best condition.

As previously discussed, the process of dry heating leads to depolymerization and oxidation of starch molecules, generating carbonyl groups. According to Oluwasina et al.,^54^ these carbonyl groups are available to establish robust hydrogen bonds with the hydroxyl groups of starch, resulting in films that are more rigid with reduced elongation. Additionally, depolymerization enhances the tendency of molecule reassociation, potentially fostering greater interactions.^11^ Consequently, the newly formed polymeric matrix exhibits distinct interactions among the starch, glycerol, and water molecules, ultimately yielding materials that are more robust. However, we can observe that the behavior is not linear, once that potato starches more depolymerized or with higher carbonyl contents do not mean that the biomembranes produced result in materials stronger. In this sense, we can observe that there is a favored size distribution of the starch molecules and the presence of functional groups to create a network with greater interaction in the matrix. Thus, the formation of the DHT_2h biomembrane was the most favored situation to result in a matrix with a network superior to that of other modified and native starches. The same behavior was discussed in the work of Lima et al.^55^ for cassava starch modified by the combination of DHT with ozone. The authors observed that depending on the grade of depolymerization, oxidation of the cassava starch resulted in hydrogels with different mechanical properties in the matrix network (weaker or stronger than the native one).

The biomembranes developed in this work showed higher resistant at break and rigid and lower flexibility when compared to films based on cassava starch modified by the same method DHT (1.79–3.63 MPa, 14.85–47.66 MPa, and 21.94–35.40%, respectively).^11^ In addition, we can observe that the effect of DHT was more expressive for the potato starch source than for the cassava starch, when comparing the performance of the biomembranes produced by these modified starches. In addition, it is noteworthy that these biomembranes display superior resistance at break and flexibility in contrast to commercially accessible collagen membranes like BioGide and CollaTape (with values of 4.8 MPa and 4.1%, respectively), widely employed in GBR applications.^56^

### 3.4 Moisture Content, Wettability, Free Surface Energy, and Biodegradability

The control biomembranes (native starch) exhibited higher moisture content compared to the modified starch biomembranes, with the impact being more visible the longer the period of DHT carried out under potato starch (Table 3). As previously discussed, subjecting starch to dry heating leads to depolymerization and oxidation (generating carbonyl groups).^54^ Additionally, depolymerization enhances the propensity for molecule reassociation, potentially fostering increased interaction.^11^ Consequently, the resulting polymeric matrix displays distinct interactions among starch, glycerol, and water molecules, contributing to the production of more resistant materials.^54^

**Table 3:** Moisture Content, Opacity, Water Contact Angle, and Free Surface Energy (γ_S_), Dispersive (γ_d_) and Polar (γ_p_) Components, and the Estimated Time to Reach 100% of Biodegradability of the Biomembranes Based on Potato Starch (Control, DHT_1h, DHT_2h, and DHT_4h)^a^

samples	moisture content (%)	opacity (%)	contact angle (°)	γ_s_ (mJ·m^–2^)	γ_p_ (mJ·m^–2^)	γ_p_ (mJ·m^–2^)	estimated time (weeks)	*R*^2^
control	18.02 ± 2.02^a^	24.12 ± 1.45^a^	27 ± 3^d^	58.0 ± 0.2^d^	44.3 ± 0.1^a^	13.7 ± 0.5^d^	4.26 ± 0.32^c^	0.98
DHT_1h	15.21 ± 1.78^a^	21.25 ± 2.10^a^	34 ± 2^c^	60.2 ± 0.6^c^	43.2 ± 0.6^a^	17.0 ± 0.8^c^	5.69 ± 0.55^b^	0.98
DHT_2h	10.11 ± 2.82^b^	11.65 ± 0.76^c^	41 ± 3^b^	65.3 ± 0.5^b^	27.2 ± 0.5^b^	38.1 ± 0.2^b^	7.00 ± 0.49^a^	0.98
DHT_4h	8.65 ± 0.95^b^	16.44 ± 0.87^b^	47 ± 2^a^	70.2 ± 0.8^a^	18.3 ± 0.4^c^	51.9 ± 0.9^a^	7.80 ± 0.45^a^	0.97

a Average ± SD (*n* = 3). a–d: Distinct letters in the same column denote a statistically significant difference among the four biomembrane compositions (Tukey test, *p* < 0.05).

Wettability and surface free energy offer insights into the interaction of the membranes with proteins, cells, and the surface of implanted materials, thereby influencing the final performance of the biomaterials. All biomembranes are hydrophilic (θ < 90°) as can be seen in , making them conducive to interactions with biological fluids, cell adhesion, and proliferation. The biomembranes based on modified starches DHT_2h and DHT_4h are less hydrophilic compared to the native counterparts, a phenomenon also noted by Liu et al.^35^ for films based on waxy potato starch modified by DHT and by La Fuente et al.^11^ for films based on cassava starch also modified by DHT. This reduction in the hydrophilicity may be assigned to changes in the molecular arrangement, resulting from the new chain conformation, as already discussed.

In relation to the surface free energy, the potato starch biomembranes produced in this study showed lower values than corn starch (63.5 mJ·m^–2^) and maize starch (70.8 mJ·m^–2^) biomembranes produced by Silva et al.^36^ and Żołek-Tryznowska and Kałuża,^57^ respectively. Overall, the dispersive component (γ_d_) exhibited significantly higher values than the polar component (γ_p_), even in the presence of polar functional groups such as hydroxyl groups. In addition, the DHT increased the surface free energy of the biomembranes: from 58.0 mJ.m^–2^ (control) to 70.2 mJ·m^–2^ (DHT_4h). An increase in the surface free energy enhances the potential of these biomembranes for use as bone biomaterials. Greater surface free energy promotes cell adhesion and spreading, stimulating apatite nucleation and bone mineralization.^58^ This, in turn, improves the bone tissue regeneration process. Prolonged DHT treatment led to a decrease in the γ_p_ component while significantly enhancing the contribution of the γ_d_ component to the overall surface free energy. This behavior corroborates the reduction in the wettability of the biomembrane based on modified starches, indicating a decrease in hydrophilic sites in the biomembranes.

The biodegradability of a biomembrane is a crucial consideration for its application as a biomaterial, ensuring compatibility with cellular processes involved in host integration and the formation of new tissues.^36^ As depicted in Figure 5A, the extent of biodegradation in the biomembranes was contingent on the duration of immersion in the cell culture medium. Notably, biomembranes based on modified starches exhibited reduction in the biodegradation, reaching around 25% after 72 h (DHT_2h and DHT_4h) of immersion when compared to native starch (∼35%). This phenomenon may be attributed to the promotion of starch–starch interactions between polymer chains in modified starches, leading to fewer available sites for interaction with the culture medium. Consequently, this results in a diminished susceptibility to biodegradation, which is interesting when you think in the application of this biomembranes as, for example, a GBR in maxillofacial defects, that should ideally exhibit a resorption time between 3 and 6 weeks.^59^

**Figure 4:**
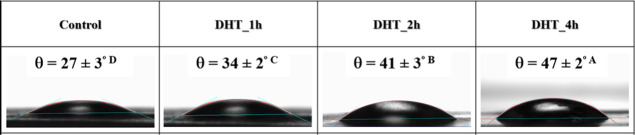
Contact angle of the evaluated biomembranes based on potato starch (control, DHT_1h, DHT_2h, and DHT_4h) with a water drop.

**Figure 5:**
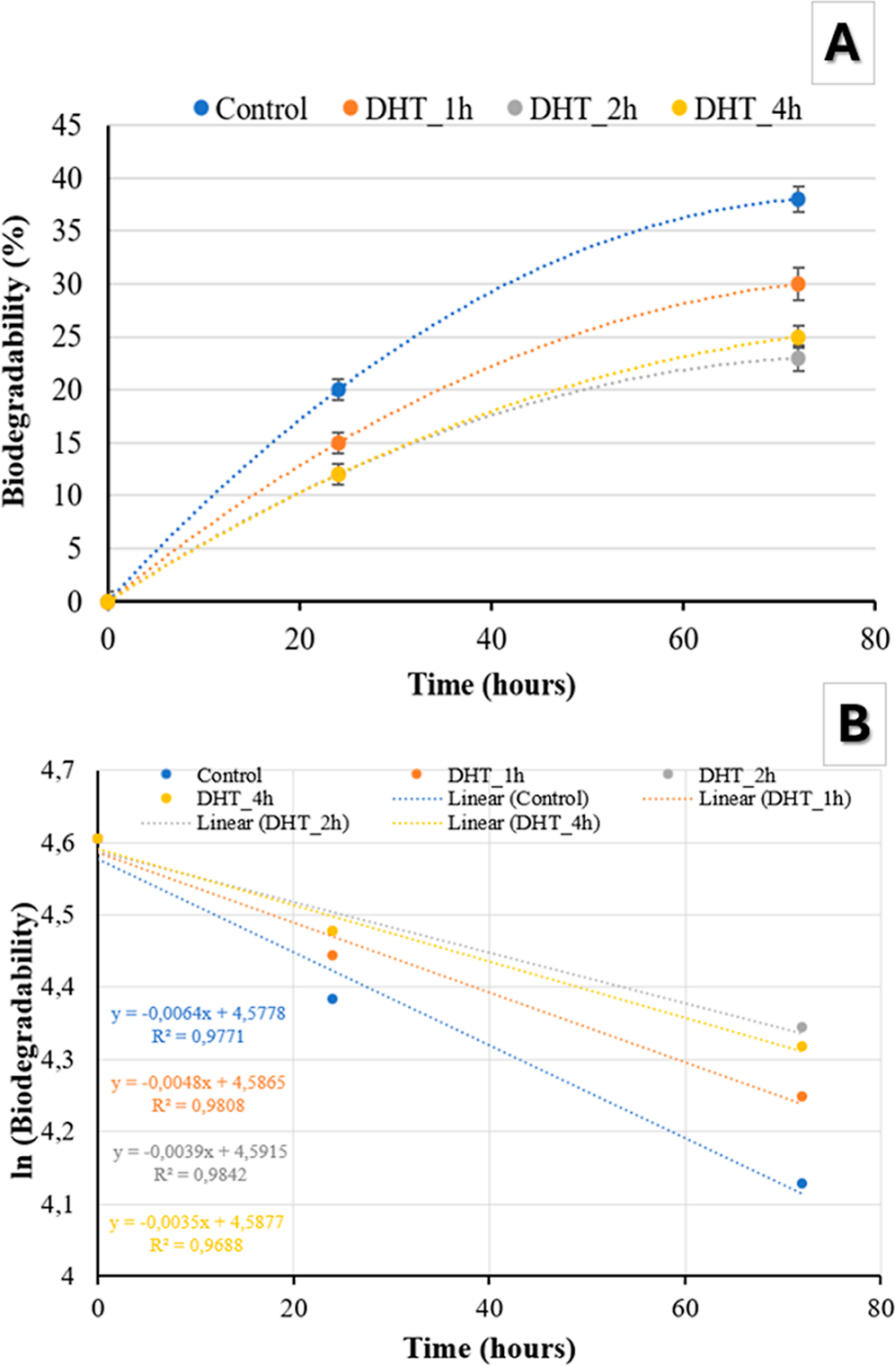
(A) Biodegradability behavior of the biomembrane for 24 and 72 h and (B) first-order reaction model used to estimate the time for total biomembrane biodegradability (*n* = 3).

The biodegradation profile was modeled using a first-order reaction model (eq 5), as shown in Figure 5B, to estimate the time required for the biomembranes to reach complete biodegradation (100%) (Table 3), which was over 4 weeks, and it is higher for DHT biomembranes, mainly for modified starch treated by 2 and 4 h (∼7 weeks). These biodegradation times align with the initial cellular events essential for osteointegration, as reported by Won et al.^100^ In this sense, DHT is an interesting alternative to improve in terms of starch biomembrane integrity to be applied as potential materials for GBR.

### 3.5 Bioactivity after Immersion in a SBF and Osteoblasts Culture

The bioactivity of the biomembranes was evaluated by assessing their ability to form a calcium phosphate layer on the surface after immersion in SBF.^60^ This process simulates the postimplantation performance of the material, which is crucial for the adhesion of proteins, and cellular signaling to initiate the cascade of events necessary for neo-bone formation.^40^ It stands as a pivotal factor for the success of a biomaterial, ensuring a robust connection between bone tissue and implant material. This connection is achieved through the establishment of a stable and intricate implant–tissue interface, essential for complete anchorage.^61^ In essence, *in vitro* tests, such as immersing synthetic materials in SBF solution, can offer valuable insights into their potential behavior *in vivo*.

**Figure 6:**
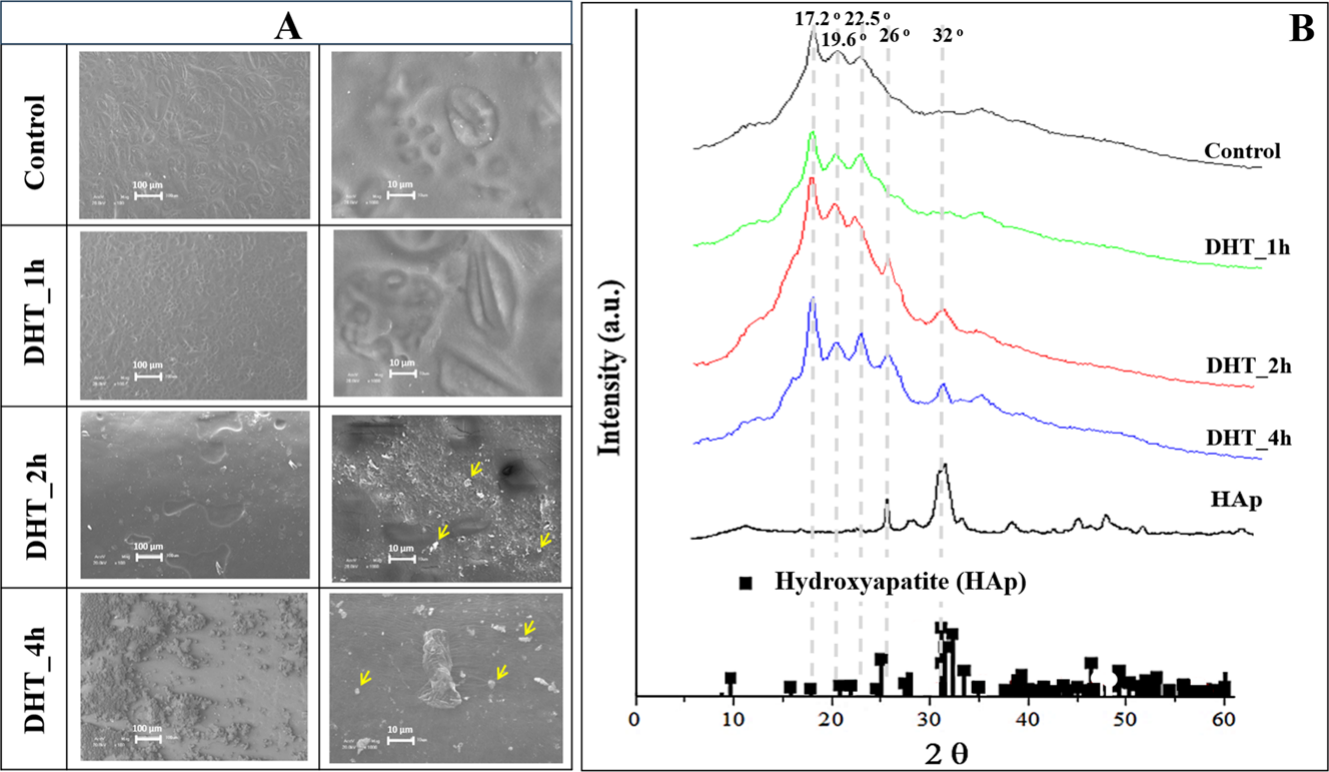
Characterization of the biomembranes following the bioactivity test conducted through immersion in SBF. (A) SEM images of the biomembranes after exposure to SBF (scale bars correspond to 100 and 10 μm in the left and right images, respectively), and (B) XRD patterns of the biomembranes exposed to SBF. The mineral phases were identified based on the diffraction patterns (■) corresponding to 9011092-hexagonal HAp from the Crystallography Open Database (COD). Yellow arrows indicate the deposition of materials on the biomembrane surfaces.

Figure 6A shows the SEM images of the biomembranes before (control) and after exposure to the SBF. Following exposure to SBF, particles with large micrometric size formed on the membrane surface. To confirm that only insoluble phosphates, like HAp, were present, the membranes were rinsed with water before analysis. The yellow arrows in Figure 6A indicate the presence of needle-like particles, typical of HAp.

XRD was carried out in order to confirm the composition of the minerals and characterize the crystallinity of the membranes. The XRD patterns illustrated in Figure 6B reveal that the biomembranes based on DHT_2h and DHT_4h, unlike the control and DHT_1h, showed prominent peaks at 26 and 32° (2θ). These peaks correspond to the hexagonal phase planes of HAp (002) and (211), respectively, as can be confirmed by comparison with the pattern 9011092-hexagonal HAp from the COD. The analysis confirms the bioactivity of the membranes and the stimulation of HAp precipitation by increasing the time of the DHT treatment.

**Figure 7:**
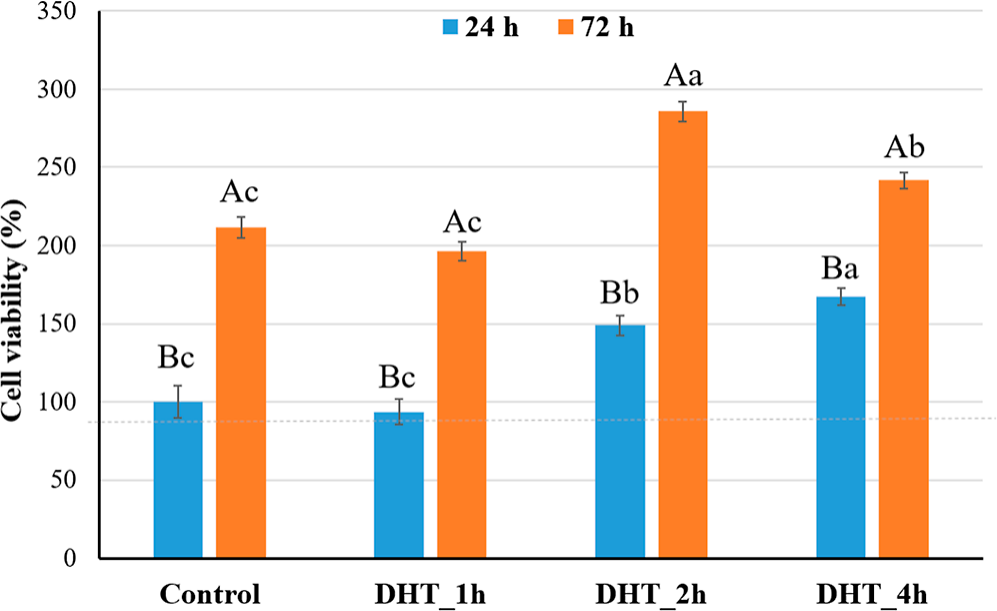
Biological performance of the biomembranes. MTT assay results for evaluating cell viability. The nonmodified starch membrane was used as control (100% cell viability after 24 h of culture). The dashed line in the figure indicates the 80% in cell viability, considered the safe limit of nontoxic materials. a, b, c: Different lowercase letters indicate a statistically significant difference among biomembrane types conditioned for the same time periods in cell culture medium (Tukey test, *p* < 0.05). A, B: Different uppercase letters denote a statistically significant difference in biomembranes of the same composition conditioned for different time periods in cell culture medium (Tukey test, *p* < 0.05).

Additionally, the toxicity of these biomembranes to osteoblastic cells was evaluated using the MTT assay. The results are depicted in the Figure 7. The biomembranes demonstrated nontoxic effects to osteoblasts as confirmed by the cell viability values close to 100%, after 24 and 72 h of culture. Moreover, the DHT treatment seems to not negatively affect the cell viability. The increased cell viability after treatment with DHT may be related to the higher hydrophilicity and higher surface energy of the membranes which stimulates cell adhesion.^62^

The results demonstrate the efficiency of DHT as a starch modification method, which is a physical technique capable of functionalizing the polymer chains, without eliciting a toxic effect.

## 4 Conclusions

This study demonstrated how the DHT and the duration of the treatment induced molecular changes, resulting in biomembranes with different physical and chemical properties. When subjected to a 2 h treatment with DHT, modified starch gave origin to membranes with a denser structure and smoother surfaces. The treatment led to heightened TS, increased rigidity, reduced flexibility, and enhanced RC, suggesting a more organized crystalline configuration compared to biomembranes derived from native starch. Moreover, biomembranes derived from modified starches, especially those exposed to DHT for 2 and 4 h, exhibited lower moisture contents and hydrophilic characteristics. They displayed higher surface free energy, diminished biodegradability, and increased bioactivity, evidenced by the formation of a calcium phosphate layer on the material’s surface after exposure to a SBF. Furthermore, in vitro evaluations did not indicate toxicity to osteoblastic cells even to biomembranes based on modified starches, which attested the safety of the process for functionalization of biopolymers aiming at medical applications.

Finally, these findings highlight the effectiveness of the DHT for a new application approach, particularly in a 2 h treatment, by increasing the potential of potato starch for producing functional biomaterial, specifically biomembranes, using a green approach.
